# Ophthalmology in the time of the Coronavirus pandemic

**DOI:** 10.22336/rjo.2021.1

**Published:** 2021

**Authors:** Mioara-Laura Macovei

**Affiliations:** * PhD, MD

Last year, in February, Romania confirmed the first case of coronavirus and on March 11, 2020, the World Health Organization declared the Coronavirus Disease 2019 a world pandemic.

The health crisis has dramatically influenced ophthalmology in our country through a significant decrease in activity. I believe the ongoing pandemic has an important and probably long-lasting collateral eye health effect.

On the one hand, at the beginning of the pandemic, in our country, as in the rest of the world, the public and the private health system was closed, all routine clinical activity, outpatient clinics, surgery, has stopped for many of us. Only the treatment of medical or surgical emergencies or pathologies that could evolve with a significant decrease or even loss of vision were allowed. In the months that followed, there was a dramatic change in the way the patient was able to access medical services with the goal of reducing the risk of transmitting coronavirus to patients or healthcare professionals. Ophthalmology is, in fact, among the specialties at risk in terms of transmitting the SARS-CoV-2 virus, especially due to the proximity of the patient during the clinical examination.

On the other hand, with all eyes on the coronavirus, people avoided hospitals during the pandemic. Many patients worried about the danger of infection with the new virus, but with severe eye damage, such as age-related macular degeneration, glaucoma, or diabetic retinopathy, giving up or delaying the necessary controls or treatments, thus risking permanent vision impairment or even blindness.

Unfortunately, in the vast majority of national or global crises, the most affected are the social categories that were previously disadvantaged. This phenomenon was also found in the case of the Covid-19 pandemic, which brought new logistical, psychological and relationship problems.

When I think about the future, I think it will be very complicated. It seems to me it is challenge to ensure an adequate number of medical services that can provide optimal ophthalmological diagnostic and treatments in the conditions of maintaining the social distance and complying with the restrictions necessary to decrease the spread of the SARS-CoV-2 virus. Waiting lists will be longer in both public and private health sectors, increasing the time necessary for the patient to access the medical or surgical treatment he needs. It will be extremely important for the entire ophthalmic community to try to reduce the effect of this pandemic on the eye health of the population as much as possible.

The pandemic also profoundly affected academic education; the sudden change in the way the classes are conducted have brought new challenges to teachers, medical students, or ophthalmology residents, all becoming dependent on a technology, sometimes harder to access and that cannot replace the valuable experience gained in training in university clinics. However, trying to balance the disadvantage of reduced or absent access to patients, there has been and still is an opportunity for study with the possibility of using many valuable online resources.

The effect of Covid-19 will last for many months or years depending on the moment the pandemic will end. Uncertainty and anxiety about the evolution of the medical crisis persists despite efforts to overcome it. During these times, it is important to stay connected, to find the humanity within us, to help each other, in the end us humans are social beings and we can only be together.

**Assist. Prof. Macovei Mioara-Laura, PhD, MD**

